# Emerging Challenges in *Salmonella* Control: The Need for Innovative and Sustainable Disinfection Strategies in Poultry Farming

**DOI:** 10.3390/pathogens14090912

**Published:** 2025-09-10

**Authors:** Nicla Gentile, Laura Lorenzo-Rebenaque, Ana Marco-Fuertes, Laura Montoro-Dasi, Clara Marin

**Affiliations:** 1 Facultad de Veterinaria, Instituto de Ciencias Biomédicas, Universidad Cardenal Herrera—CEU, CEU Universities, Alfara del Patriarca, 46115 Valencia, Spain; nicla.gentile@uchceu.es (N.G.); ana.marcofuertes@uchceu.es (A.M.-F.); laura.montoro@uchceu.es (L.M.-D.); 2 Institute of Science and Animal Technology, Universitat Politècnica de Valencia, 46022 Valencia, Spain

**Keywords:** biosecurity, C&D, bacteriophages, essential oils, positive biofilm, nanoparticles

## Abstract

*Salmonella* is one of the primary causes of foodborne infections worldwide and is often linked to the consumption of poultry products. Despite the implementation of numerous control programmes, the persistence of *Salmonella* in poultry environments remains a challenge, exacerbated by the emergence of strains resistant to traditional disinfectants. This review examines the key factors associated with the limitations of disinfection and the new strategies employed in poultry production, underscoring the need for more sustainable and effective alternative solutions. Various chemical (nanoparticles), physical (ultraviolet light, heat, pressurised steam, infrared radiation) and biological (bacteriophages, essential oils, and positive biofilm) treatments are examined. Of the various alternatives assessed, some have shown promising antimicrobial activity against *Salmonella* in vitro and under experimental conditions. However, their application in real-field settings is still limited, and few studies evaluate their effectiveness on a commercial scale. The review emphasises the importance of integrating these alternatives within broader biosecurity programmes, supported by clear regulations to minimise the risk of transmission. In conclusion, the adoption of innovative and sustainable approaches, combined with strengthened biosecurity measures, represents a key strategy to reduce *Salmonella* contamination in poultry farms, protect public health and promote responsible production systems.

## 1. Introduction

Worldwide, *Salmonella* has been recognised as one of the leading causes of bacterial gastroenteritis related to the consumption of animal products, particularly those derived from the poultry industry [1]. Over time, different biosecurity practices have been implemented to reduce the prevalence of *Salmonella* in poultry farms and to protect consumers from associated health risks [1,2]. Among these, the implementation of *Salmonella* National Control Programmes (SNCP) in Europe marked a significant milestone in the effective control of the bacterium [3]. Due to the implementation of SNCP measures, from 2007 to 2023 the prevalence of the most significant serovar for human health gradually decreased in breeding *Gallus gallus*, laying hens and broilers. However, in the short-term trend from 2019 to 2023, the downward trend in *Salmonella* cases plateaued, with prevalence remaining relatively stable between 2019 and 2023 [4]. In the latest report published by the European Food Safety Authority (EFSA) [4], which includes data from 2023, a significant increase in human gastroenteritis cases was observed, with 12,278 more cases reported compared to previous years (total human salmonellosis cases in 2023: 77,486), underscoring the critical challenges faced by the poultry industry [4].

The serotypes of *Salmonella enterica* commonly associated with human infections are *S. enteritidis*, *S. typhimurium* and the monophasic variant of *S. typhimurium* (1,4,[5],12:i:-) [4]. Moreover, since 2014 the serotype *S. infantis* has emerged as the third most prevalent associated with human illness related to the consumption of chicken meat [1,3]. Infection generally leads to a self-limited gastroenteritis, although certain serovars can cause severe conditions, such as Reiter’s Syndrome or Typhoid Fever, particularly in children and elderly people, who are considered high-risk populations for this infection [5]. However, one of the main issues associated with these bacterial species is their ability to acquire genetic traits that enhance their resistance and persistence in the environment, such as biofilm formation [6,7,8]. Sub-lethal exposure and biofilm formation have been shown to favour the persistence of resistant populations, compromising the effectiveness of conventional cleaning and disinfection (C&D) [6,9,10]. The biofilm is a complex three-dimensional structure that develops through a gradual and dynamic process, providing bacteria with high protection against external agents while simultaneously hosting various microorganisms, thereby acting as a reservoir of pathogens and facilitating the transmission of genes associated with virulence and resistance to drugs and disinfectants, ultimately increasing the overall health risk [6,7,8]. Indeed, it cannot be ruled out that, over time, new serotypes may emerge with innovative genetic characteristics, capable of developing increasingly resilient survival mechanisms against adverse environmental conditions [4]. This situation is further compounded by the adoption of less intensive farming systems. Although extensive systems have many benefits in terms of animal welfare, they are associated with an increased risk of pathogen exposure [2].

Numerous scientific studies highlight the complexity of controlling and eliminating *Salmonella* in poultry farms. However, this task is becoming increasingly challenging: on the one hand, we must address the new genotypic and phenotypic traits developed by the bacterium; on the other, we must respond to society’s growing demands for greater environmental sustainability. In light of this scenario, the key question is: What tools do we currently have at our disposal to effectively combat *Salmonella*?

Disinfectants such as glutaraldehyde, quaternary ammonium compounds (QAC), and formaldehyde have been used in the field for years, and, despite their strong antibacterial capacity, they are prone to developing resistance [3,9,11,12,13,14,15,16,17,18]. Among these, chlorine-based products are particularly popular due to their broad commercial availability, effectiveness, and low cost [7,19]. In addition, the use of formaldehyde, despite being one of the most effective antibacterials, has been banned in livestock farming according to the European Regulation 605/2014 due to its high toxicity to human health [3]. Moreover, in addition to the environmental and safety concerns associated with their use, these disinfectants may be ineffective in the presence of organic matter or biofilm, allowing only partial removal of the microbial community on surfaces [19,20]. As a result, this situation highlights the urgency of identifying new control measures that are more effective, sustainable and safe for humans and animals, tailored to the characteristics of these new strains [1]. This need is further reinforced by increasing regulatory pressure aimed at reducing the use of chemical disinfectants to minimise environmental impact, improve animal welfare and protect consumer health [20].

In this context, the study aims to examine innovative and eco-friendly methods for C&D poultry farms, with a focus on controlling zoonotic *Salmonella* strains and compliance with current European regulations. Additionally, biosecurity measures will be analysed, with particular attention to external measures, which are essential for preventing the introduction and spread of pathogens within poultry farms.

## 2. Methodology

An in-depth literature review was conducted to analyse C&D protocols applicable to the poultry sector for the control of *Salmonella*. The bibliographic search was carried out in PubMed and Google Scholar, and included only articles published in English. The main keywords used, in various combinations, were as follows: *Salmonella*, biosecurity, poultry farm, cleaning and disinfection, chemical disinfection, physical disinfection, bacteriophage and essential oil.

Articles published between 2011 and 2025 were considered, with the exception of two studies from 1985 and 2000, which were included to clarify basic concepts. The inclusion criteria were: (i) evaluation of the efficacy of chemical, physical, biological or combined C&D methods; (ii) studies conducted in laboratory, pilot plant or field conditions relevant to poultry farming; and (iii) outcomes related to *Salmonella* control or microbial load reduction. Exclusion criteria were: (i) studies not relevant to poultry production; and (ii) publications focused exclusively on pathogens other than *Salmonella*.

Particular attention was given to studies evaluating innovative C&D protocols and their potential for practical application. These studies were systematically categorised into chemical, physical and biological methods, as well as their combinations, in order to highlight strengths, limitations, and future research perspectives.

## 3. Emerging Challenges in *Salmonella* Control

As previously mentioned, a growing obstacle in the fight against *Salmonella* in poultry farms is its progressive resistance, or more accurately, “tolerance”, to commonly used disinfectants and sanitisers [9]. Recent studies have shown that *Salmonella* can adapt to and survive exposure to compounds such as oxidants, acids (both organic and inorganic), phenols and surfactants, particularly under environmental conditions that reduce their efficacy [9]. It is, however, essential to distinguish between the concepts of resistance and antimicrobial tolerance. In the case of antibiotics, the EU and other regulatory bodies establish standardised cut-off values that make it possible to precisely determine when a microorganism can be considered resistant [9]. In contrast, for disinfectants, such thresholds do not exist; in this context, the ability of microorganisms to survive or adapt to sub-lethal concentrations of these compounds is instead defined as antimicrobial tolerance [9,10].

In several poultry production settings, such as hatcheries or battery cage systems, the exposure of microorganisms to non-lethal concentrations of disinfectants may favour the selection of strains with acquired resistance to conventional treatments [21]. Moreover, pathogens in poultry environments are constantly exposed to environmental fluctuations (temperature, humidity, pH), which may induce stress conditions capable of triggering survival mechanisms. These include adaptive mutations, such as biofilm formation, or the transfer of mobile genetic elements, for instance plasmids carrying resistance genes [9,22]. Under such conditions, disinfectants may act in a biostatic manner, temporarily inhibiting bacterial growth without completely eliminating the microorganisms [9].

This phenomenon can generate a vicious cycle; it is therefore recommended that different production cycles employ disinfectants with diverse modes of action or integrate alternative approaches in order to reduce the ability of pathogens to adapt and develop tolerance [10].

## 4. Disinfection Effectiveness: Key Factors and New Approaches

With the development of intensive and large-scale poultry farming, environmental disinfection has become particularly important, and its effectiveness depends on the performance of the disinfectants, as well as their synergistic action with the applied detergents, the treated surface and the method of application [11].

### 4.1. Surface

Among the factors that can influence the effectiveness of C&D protocols, surface type plays a critical role. Porous, irregular and smooth surfaces (such as glass, plastic or stainless steel) pose varying levels of challenge for effective C&D. Several studies support these observations, including Jang et al. [23], who evaluated the effectiveness of various commercial chemical disinfectants against *S. typhimurium*, considering the type of surface (wood and stainless steel) and the application temperature. The results showed that, for most disinfectants tested, bactericidal efficacy was significantly reduced at low temperatures. Moreover, bacterial loads on wood were more challenging to eliminate than those on stainless steel, confirming that *Salmonella* can lodge in surface pores, making complete removal challenging [23]. Similarly, Megahed et al. [24] demonstrated that the effectiveness of disinfectants in eliminating *Salmonella* varies greatly depending on the type of surface (plastic, nylon, rubber and wood), showing that smooth surfaces can be decontaminated more easily, even in the presence of organic matter. Corcoran et al. [25] evaluated the effectiveness of three commonly used disinfectants (sodium hypochlorite, sodium hydroxide and benzalkonium chloride) in removing experimentally induced biofilms from concrete surfaces after 48 and 168 h of maturation [25]. The degree of maturation of this structure can significantly influence the effectiveness of disinfectants. In fact, the results of the study show that only sodium hydroxide was able to completely eradicate the 48 h old biofilm, while none of the disinfectants were effective against the 168 h biofilm, which had developed a more compact, adherent and resistant structure. These findings highlight the importance of timely intervention during the early stages of biofilm formation, before structural and physiological characteristics emerge that increase resistance to chemical treatments [25].

### 4.2. Water Quality

Another factor that can affect the effectiveness of disinfectants is the quality of the water, such as hardness or pH. One study found that the sanitising activity of iodophors is significantly reduced at pH values above 8.0, with an even more pronounced effect in the presence of hard water [26]. Another study examined the influence of water source (tap water vs. groundwater) on the production and sanitising efficacy of slightly acidic electrolysed water (SAEW). Results showed that the hardness of the input water is a key factor in the production of effective SAEW. Tap water, with a hardness of 29 ppm, proved more suitable for producing effective SAEW than groundwater, which had a hardness of only 12 ppm. However, low water hardness can be compensated by adding a combination of hydrochloric acid and salts, thus improving the effectiveness of the electrolysis process [27].

### 4.3. Temperature

Temperature is another important factor, as some disinfectants may lose effectiveness at low temperatures or evaporate too quickly at high temperatures, reducing the required contact time for effective disinfection. For example, in the recent study conducted by Kroft et al. [28], it was observed that peracetic acid (PAA), commonly used in the poultry industry to reduce *Salmonella* on carcasses, varies in effectiveness depending on concentration and application temperature. Indeed, the results showed that at the same concentration, PAA was more effective at 4 °C than at 27 °C [28].

**Figure 1:**
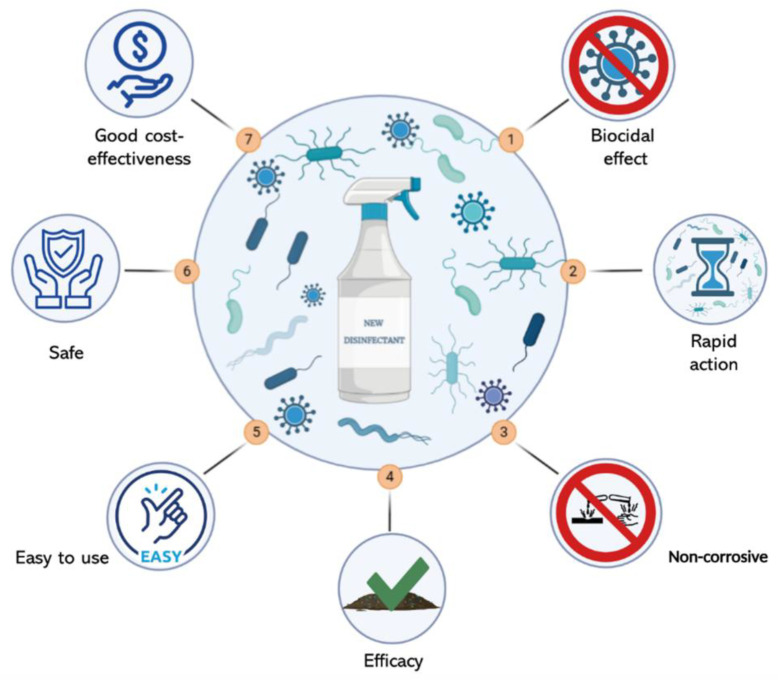
Schematic representation of the key features for the development of new disinfectants (created by Biorender).

In this context, the future goal is to create new disinfectants that meet the basic requirements: (1) a pronounced biocidal effect; (2) acting within a short period of microorganism exposure; (3) being non-corrosive to metals and not damaging other materials present on the objects to be treated; (4) maintaining their efficacy even in the presence of organic substances; (5) being easy to use (e.g., dissolving easily, with a long shelf life, and environmentally friendly); (6) being safe and non-allergenic for humans and animals and environment; and (7) providing a good cost-effectiveness ratio, including the equipment necessary for disinfection (Figure 1) [29].

The following summary outlines the new approaches adopted for *Salmonella* control, categorised into chemical, physical, chemical-physical and biological methods, with an indication of the related methodologies applied and the bibliographic sources referenced (Table 1) that will be discussed in detail in Section 5.

**Figure 2:**
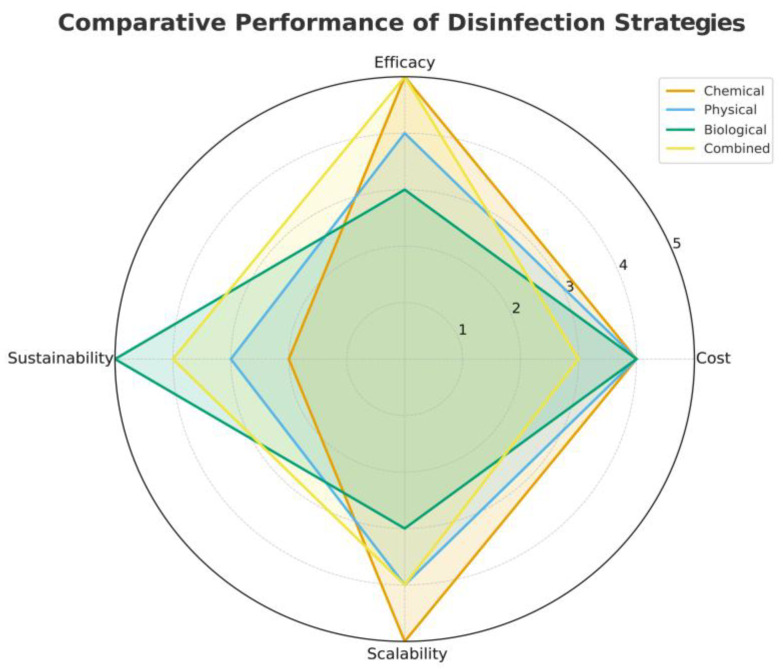
Comparative radar chart of chemical, physical, biological and combined approaches, evaluated across four criteria (efficacy, cost, scalability and sustainability) on a scale from 1 (very dissatisfied) to 5 (very satisfied).

To visualise the differences among the main C&D approaches analysed in this review, a radar chart (Figure 2) was developed to compare chemical, physical, biological and combined methods across four key criteria: efficacy, cost, scalability, and sustainability.

## 5. Next-Gen Solution

### 5.1. Chemical Methods or Their Combination

The increasing spread of resistant strains, together with regulatory restrictions on the use of chemical disinfectants, is driving research towards the development of new alternative formulations or the combined use of different chemical treatments. These strategies may prove particularly effective in contexts where traditional methods show reduced efficacy [12,14]. However, they continue to be widely used due to their proven efficacy and cost-effectiveness. This section examines in vivo and in vitro studies on the use of chemical methodologies in surface disinfection processes.

In this context, the bactericidal activity and toxicity of a combination of a quaternary ammonium surfactant with other disinfectants (chlorhexidine acetate and glutaraldehyde) were evaluated by Chen et al. [11] with the aim of developing a new broad-spectrum disinfectant. QACs are widely used in the poultry industry; however, in the presence of organic matter, they show their vulnerability [11]. Therefore, the next step is to assess new formulations capable of overcoming this limitation. In this context, their results revealed that the combination of the ammonium salt N-dodecyl-2-(pyridin-1-ium) acetamide chloride with both disinfectants appears to be safe and capable of meeting microbial reduction requirements, with its effectiveness not being influenced by the presence of organic matter. However, data on its efficacy against *Salmonella* are lacking, and no field testing has been conducted yet.

Another innovative alternative gaining increasing attention is the use of silver or gold nanoparticles (AgNP or AuNP), which are already widely employed in healthcare for their proven antibacterial properties [57]. Recently, their potential has expanded beyond healthcare, generating growing interest in their use for disinfection in various sectors, including poultry [30,58]. Nanoparticles can be synthesised using different methods (chemical and biological) and used either alone or in combination with other compounds to optimise their antimicrobial effects [58]. For example, Disa de Emery et al. [30] analysed the effectiveness of AgNPs (synthesised chemically) as a disinfectant for polyethylene surfaces against *S. enteritidis*. After just 30 min of contact, the AgNPs caused a significant bacterial reduction (3.91 log_10_ CFU/cm^2^), higher than that achieved with a conventional disinfectant based on polyhexamethylene biguanide hydrochloride combined with QACs (2.57 log_10_ CFU/cm^2^) [30]. However, the effectiveness of AgNPs increases even further when combined with a reducing agent, such as calcium hypochlorite [Ca(OCl)_2_], as demonstrated by Mohammed et al. [29]. The advantages of chemical synthesis of AgNPs are relatively simple, low-cost and offer high yields [58]. Nevertheless, chemical synthesis involves the use of reagents that can be potentially harmful to the environment [58]. In this context, the biological synthesis of nanoparticles represents a sustainable alternative, although it generally yields lower amounts [58]. Recent studies have reported promising results in terms of antibacterial activity against both Gram-negative (G−) and Gram-positive (G+) bacteria, highlighting the potential of biologically synthesised nanoparticles as a more eco-friendly solution [59,60,61].

### 5.2. Physical Methods or Their Combination

The physical methods or their combination involve the use of two or more physical techniques in a complementary way to enhance disinfection or pathogen control. This approach leverages the synergistic effects of methods such as ultraviolet (UV) light, heat, pressurised steam, etc. These strategies are particularly useful in contexts where the use of chemical substances is limited or when non-toxic and residue-free disinfection is required. This section examines various physical methods to optimise disinfection processes. However, although promising as methods, they require a high energy expenditure, and the costs often limit their large-scale application, which reduces their overall feasibility in commercial farms despite their proven efficacy [20].

UV light as an antimicrobial alternative has gained increasing interest, especially following its approval by the Food and Drug Administration (FDA) in 1997 for microbial control in meat-based products [31]. Today, UV radiation is widely applied for surface decontamination; however, its antimicrobial efficacy can be further enhanced when combined with other technologies, such as light-emitting diodes (LEDs). The advantage of this combined approach is that, unlike traditional mercury-based UV lamps, UV-LEDs are more environmentally friendly, durable and energy-efficient. A relevant study conducted by Calle et al. [31] evaluated the effectiveness of UV light in combination with LEDs at two intensities (2 mW/cm^2^ and 4 mW/cm^2^) on stainless steel and high-density polyethylene surfaces contaminated with a cocktail of five *Salmonella* strains (6.0 log_10_ CFU/cm^2^). The results showed that after only 60 s of exposure at 4 mW/cm^2^ the stainless steel surfaces had recorded reduction of 3.48 log_10_ CFU/cm^2^. High-density polyethylene surfaces also showed a significant reduction, albeit slightly lower, confirming the effectiveness of UV-LED technology in controlling bacteria on different surfaces.

Another technology that utilises UV rays is photohydroionization, developed by NASA to reduce and neutralise indoor air pollutants; this technology uses high-intensity UV-C light (100–280 nm) directed at a surface [32]. Although it represents a valid alternative, it has proven to be more effective against viral pathogens than against bacteria. In the study by Appel et al. [32], photohydroionization was evaluated for disinfecting poultry litter in vitro contaminated with bacterial, fungal and viral agents. The results showed a total reduction in viruses (100%), while partial reductions were observed for bacteria: 82.3% for enterobacteria and 50% for total bacterial count. These findings suggest that although photohydroionization is less effective against bacteria, it still represents a promising method for combating viral contamination in poultry farms. It is likely that, when combined with other methodologies, it could achieve even more effective results.

Heat also represents an effective physical method for the elimination of pathogens and can be applied through various means, including infrared radiation [33]. These rays can warm the surrounding environment through radiation, without the need for a physical medium to transfer heat. Infrared radiation, with wavelengths ranging from 2 to 10 µm, has recently been used for the disinfection of agricultural products. An innovative application of this technology involved the treatment of poultry litter made of rice husks, widely used in poultry farms in Asian countries, experimentally contaminated with *S. typhimurium* at a concentration of 10^7^ CFU/g. For this purpose, an automated device was developed to apply infrared radiation for exposure times ranging from 1 to 3 min. The results showed that a treatment at 120 °C for 3 min reduced the bacterial load by approximately 10^4^ CFU/g, confirming the effectiveness of the method [33]. However, the penetration capacity of infrared radiation is limited and poorly suited for porous surfaces [62]. Moreover, it is important to highlight that in Europe, litter recycling is not a permitted practice, both due to regulatory constraints and the challenges in ensuring the hygienic and sanitary safety of the treated material [63].

Finally, the last method identified consists of using pressurised steam for surface disinfection, combined with hot air. Steam is characterised by a higher heat transfer coefficient than hot water. On the other hand, hot air helps eliminate residual moisture, which could favour the growth of microorganisms [64]. In this line, Reina et al. [34] evaluated the effectiveness of pressurised steam followed by forced hot air for cleaning broiler transport container floors contaminated with *S. infantis* and other *Enterobacteriaceae* [34]. The study compared six different treatment protocols applied to both fibreglass and plastic surfaces, using traditional cleaning methods as a reference. The results showed that the combined use of steam (7.2 bar) followed by hot air (171 °C) significantly reduced *Salmonella* levels. In particular, the fibreglass surfaces showed lower bacterial counts (0.06 log_10_ CFU/cm^2^) compared to the plastic surfaces (0.42 log_10_ CFU/cm^2^). These findings highlight not only the strong antimicrobial potential of the combined steam and hot air treatment but also the influence of surface material on disinfection efficacy.

### 5.3. Chemical/Physical/Biological Combination

This section explores how combining different disinfection methods can enhance the overall effectiveness of the process. Such integrated strategies are particularly valuable in contexts where the application of a single method is insufficient to ensure complete pathogen elimination. Nevertheless, they present limitations related to costs, operational complexity and their potential applicability on a large scale. These methods include combined treatments of ultraviolet light (UV)—light-emitting diodes (LEDs) with slightly acidic electrolysed water (SAEW), peroxyacetic acid (PAA) or lactic acid bacteria (LAB); thermal treatments combined with chemical disinfectants; and methods that exploit physicochemical properties, such as plasma-activated water and electrochemically activated water.

SAEW, characterised by a pH between 5.0 and 6.5, is produced through the electrolysis of a low-concentration sodium chloride solution in the presence of diluted hydrochloric acid, generating hypochlorous acid (HOCl). Among the main advantages of this method are the potential to minimise the escape of chlorine gas and the impact on human health, contributing to “green disinfection”. For these reasons, SAEW represents a valid alternative to traditional chlorine-based treatments. A study conducted by Zang et al. [35] on the use of SAEW in combination with UV light was evaluated for decontaminating *S. enteritidis* on plastic poultry transport cages and other common surfaces in farms. Previous studies have already reported the use of SAEW to reduce the microbial load on poultry farm surfaces [65,66]. The results showed that SAEW alone and in combination with UV significantly reduced *S. enteritidis* populations compared to treatment with composite phenol, despite the presence of organic matter. In cell suspension tests, the combination of UV and SAEW at 70 mg/L of available chlorine eliminated detectable *S. enteritidis* after 80 s. The most significant surface decontamination was observed on glass, with a reduction of 3.64 log_10_ CFU/cm^2^, while rougher surfaces showed less reduction, likely due to stronger faecal matter adhesion [35]. Byun et al. [19], instead, investigated the combination treatment of PAA or LAB with UV-C against *S. enteritidis* biofilms formed on stainless steel, silicone rubber and ultra-high molecular weight polyethylene. Currently, PAA can be applied at a maximum concentration of 200 μg/mL and is preferred over chlorine for its acidic and oxidising properties. LAB, on the other hand, can be used at a concentration of 2–5%, and its mechanism of action is based on reducing intracellular pH and breaking the proton concentration gradient across the inner cell membrane. In this particular study, different concentrations of PAA and LAB were used and applied for 5 min, while radiation emitted by UV light was at 253.7 nm for 5 and 10 min at room temperature. The results show that the single treatment reduces the concentration of *Salmonella* on the contact surface, but the combination of treatments proves to be more effective. In fact, the combination of PAA with UV-C reduces the biofilm 3.65–6.19 log_10_ CFU/cm^2^ on stainless steel, 3.85–6.10 log_10_ CFU/cm^2^ on silicone rubber and 3.10–6.00 log_10_ CFU/cm^2^ on ultra-high molecular weight polyethylene. Instead, for the LAB with UV combination, the range biofilm reduction was 4.06–6.19 log_10_ CFU/cm^2^ on stainless steel, 3.35–6.10 log_10_ CFU/cm^2^ on silicone rubber and 3.58–6.00 log_10_ CFU/cm^2^ on ultra-high molecular weight polyethylene [19].

Among thermal treatments combined with chemical disinfectants, Ohashi et al. [36] analysed the ability of different *Salmonella* strains to form biofilms and, in order to better understand their resistance, developed an in vitro experimental model using ceramic and stainless steel supports, materials that closely mimic the typical surfaces found in farming environments. Through this model, the researchers evaluated the effectiveness of various disinfection methods. The results showed that traditional treatments, such as the use of surfactants at temperatures between 25 and 65 °C, or the application of QACs or hypochlorous acid, were not sufficient to eliminate the pathogen. In contrast, a combined strategy consisting of treating the surfaces with surfactants at 65 °C, followed by rinsing with water at 80 °C and subsequent application of chlorine dioxide and dolomitic lime, proved to be much more effective, significantly reducing the number of persistent bacterial cells. According to the authors, this approach could also be practically applied in real farming settings [36]. Similar results were observed in the study conducted by Šovljanski et al. [37], which aimed to evaluate the synergistic effect of heat and PAA. The findings showed that combining 1.5% PAA with a temperature between 60 and 65 °C and a contact time of 3 min completely eliminated *Salmonella*, regardless of the initial level of contamination [37]. In this case as well, the combination of heat and disinfectants proved to be a promising strategy for controlling the pathogen.

Other treatments that combine chemical and physical properties are plasma-activated water and electrochemically activated water. The former is obtained by exposing water to a high-voltage atmospheric cold plasma, a process that generates various oxygen radical species responsible for its biocidal activity. This newly introduced technology has been applied for the decontamination of surfaces and food [38]. Instead, electrochemically activated water is produced using water, salt and electricity through a membrane electrolysis process, leading to the formation of HOCl. Its mechanism of action is based on disruption of the bacterial cell wall, causing the loss of bacterial DNA inside the cell [39]. Studies on the effectiveness of these technologies have been conducted by Měřínská et al. [38], who evaluated the efficacy of plasma-activated water against *Salmonella* inoculated on surfaces commonly found in poultry houses, such as stainless steel, PVC, wood and concrete. Each surface was experimentally contaminated with a cocktail of *Salmonella* strains at a 7–8 log_10_ CFU concentration. The results showed a significant bacterial reduction, bringing it below the detection limit within 30 s of treatment. However, for porous and rough surfaces like wood and concrete, a longer treatment time was necessary [38]. On the other hand, the study conducted by Wilsman et al. [39] evaluated the antibiofilm activity with electrochemically activated water, monitoring the biofilm of *S. heidelberg* on stainless steel and polyethylene surfaces. The results of the study showed that electrochemically activated water could reduce the biofilm by up to 2.67 log_10_ CFU/cm^2^ but was not able to eliminate it completely, and it proved to be more effective on stainless steel surfaces compared to polyethylene. However, it did not show significant differences compared to common disinfectants [39].

Finally, a robotic system integrated with Internet of Things (IoT) technology has recently been developed for litter sanitation, combining decontamination through ozone and UV light. Both technologies offer significant advantages in reducing microbial load. The robot is capable of monitoring the sanitary condition of the litter in real time and automatically activating the decontamination process. This innovative approach helps to reduce the farmer’s direct exposure to chemical disinfectants [40].

### 5.4. Biological Methods

#### 5.4.1. Bacteriophages

Alternative biological solutions are under development and are starting to be used in animal production [20]. In recent years, bacteriophages (or phages) have been considered as an alternative to conventional disinfectants in C&D protocols, showing promising results [3,7]. This method, known as phage-based biocontrol, represents an ecological and natural technology that enables the elimination of pathogenic microorganisms without harming beneficial ones [7,67]. Bacteriophages, in fact, are natural agents that are environmentally safe. Currently, the phages used in biocontrol products are all of wild origin; they are isolated from natural sources and are specifically associated with a particular bacterial strain type [7,67]. Most phage-based preparations do not contain added chemicals, but only an aqueous solution of phages and a low concentration of salts, and have relatively low production costs [67].

Currently, few studies document the effectiveness of using bacteriophages as biocides in the poultry industry, but the available results are very promising. A significant example is the study by Sevilla Navarro et al. [3], which evaluated the use of a cocktail of lytic bacteriophages (vB_Si_CECAV_FGS009; vB_Si_CECAV_FGS017; vB_Si_CECAV_FGS029; and vB_Si_CECAV_FGS030) as a biocide, in combination with C&D protocols, in 10 commercial poultry farms in northern Spain. The protocol involved applying the phage cocktail twice, with a 24 h interval between C&D, at a concentration of 10^8^ PFU/mL. The results showed that this double application of phages reduced *Salmonella* contamination in the farms by 100% [3]. Moreover, Korezeniowski et al. [41], evaluated the efficacy of individual bacteriophages (UPWr_S1, UPWr_S3, UPWr_S4) and their combination (UPWr_S134) in controlling biofilms formed by two strains of *S. enteritidis* (327 lux and ATCC 13076) in vitro. Tests were conducted using 96-well microplates, stainless steel surfaces and artificially contaminated poultry drinkers. The results showed that *S. enteritidis* ATCC 13076 had a biofilm-forming ability 2.8 times greater than the 327-lux strain, but both strains were susceptible to phage treatments. However, the UPWr_S134 cocktail demonstrated greater effectiveness in reducing biofilm, with up to 98% reduction rates on 96-well microplates at high titres (10^9^ PFU/mL). Similar results were obtained from tests on stainless steel surfaces. Finally, in the drinker experiment, treatment with the phage cocktail inhibited the growth of *S. enteritidis* 327 lux in contaminated water, unlike in the untreated drinkers [41]. In this line, Gvaladze et al. [42], evaluated the efficacy of a commercial phage product containing six phages (vB_SalS_OBO18, vB_SalM_RMS3b, vB_SalS_RMP9, vB_SalM_MP82, vB_SalM_TAT2F, vB_SalM_DIN2) against various *Salmonella* serotypes isolated from poultry products on stainless steel surfaces. Two experiments were conducted: the first involved applying the phage cocktail to the surface contaminated with *S. enteritidis* before drying, and the second involved applying the phage cocktail to the surface contaminated with *S. enteritidis* and a mixture of five serotypes after complete drying. The results indicated that the fresh contamination led to a greater reduction in *Salmonella*, with bacterial levels below the detection limit two hours after treatment. In the second experiment, the serotype mixture showed a reduction of up to 2.4 log_10_ after 4 h of treatment [42]. Rogovsk et al. [43] examined the efficacy of a lytic bacteriophage isolated from pig manure (SM1) in controlling *S. enteritidis* in poultry litter. Two protocols were tested: one involved a single treatment, while the other consisted of repeated applications of phage SM1 (10^6^ PFU/mL) after 6 and 12 h. Specifically, litter initially negative for *Salmonella* was contaminated with 10^5^ CFU/mL of *S. enteritidis*. The results showed that the repeated application of phage SM1 reduced *Salmonella* counts by over 99.9%. Additionally, it is noteworthy that the phage remained active in the poultry litter for over 35 days [43]. Finally, Ge et al. [44] isolated a lytic phage from poultry faecal samples and evaluated its effectiveness against *S. enteritidis* on metal surfaces. The experiment involved contaminating a metal surface with *S. enteritidis* at a concentration of 1 × 10^6^ CFU/mL, followed by the application of the phage at a concentration of 1 × 10^10^ PFU/mL, resulting in an initial inoculum of 1.0 × 10^8^ PFU/cm^2^. The results showed a reduction in the bacterial load of *S. enteritidis* by 0.951 log_10_ CFU/mL [44].

These results pave the way for a new eco-sustainable strategy. One potential limitation is the emergence of bacteriophage-resistant bacterial variants, which could hinder long-term effectiveness [3,68]. To address this issue, the use of autophages, as proposed by Ge et al. [44], could offer a solution, since the prolonged coexistence of bacteria and phages in the same environment promotes coevolutionary dynamics. Moreover, being specific to certain bacterial species, no negative impact on the surrounding ecosystem is expected [3].

#### 5.4.2. Essential Oils

Essential oils (EOs) and plant extracts are showing great potential for their antimicrobial properties [69]. Interest in their use has grown particularly due to the increasing antibiotic resistance, which has made infection treatments more challenging over time [70]. These are mixtures of aromatic compounds extracted from various parts of plants, such as fruits, roots, rhizomes, leaves, flowers, bark, buds, twigs, wood and seeds, known for their distinctive fragrances [69]. EOs are produced by over 17,500 plant species, but only about 300 of these are commercially available. Humans have been using EOs for thousands of years, not only as ingredients in perfumes or as flavouring agents in food, but also in traditional medicine, due to their numerous biological properties, including antimicrobial effects [44]. However, in vivo studies assessing their applicability under real farming conditions are still lacking. Most of the available research has focused on testing the activity of essential oils in vitro against pathogens of relevance to the poultry sector.

In this regard, several in vitro studies have demonstrated the promising efficacy of EOs. For example, Olawuwo et al. [52] investigated the in vitro antibiofilm activity of plant extracts from *Acalypha wilkesiana*, *Alchornea laxiflora*, *Ficus exasperata*, *Jatropha gossypiifolia*, *Morinda lucida*, and *Ocimum gratissimum* against major poultry pathogens, including *Salmonella* spp. Among the tested extracts, the acetone extract of *M. lucida* demonstrated the best antibiofilm effect against *S. enteritidis* [52]. Similarly, Wang et al. [46] evaluated the antibacterial potential of the EOs thymol, carvacrol, citral, cinnamaldehyde, limonene and β-pinene against *S. enteritidis* by determining their minimum inhibitory concentrations (MIC). The results showed that thymol (128 μg/mL), carvacrol (256 μg/mL) and cinnamaldehyde (128 μg/mL) were particularly effective in inhibiting *S. enteritidis* biofilm formation [46]. Ebani et al. [50] investigated the in vitro antimicrobial activity of EO extracted from *Aloysia triphylla*, *Cinnamomum zeylanicum*, *Cymbopogon citratus*, *Litsea cubeba*, *Mentha piperita*, *Syzygium aromaticum*, and a 1:1 mixture of *S. aromaticum* and *C. zeylanicum* against *S. enteritidis* and *S. typhimurium* strains previously isolated from poultry. The results showed the highest antibacterial activity for *C. zeylanicum*, with MICs ranging from 1.26 to 0.63 mg/mL, followed by *S*. *aromaticum* (MICs from 2.637 to 0.164 mg/mL) and the mixture of the two oils (MICs from 1.289 to 0.322 mg/mL) [50]. Hassanzadeh et al. [47] evaluated not only the in vitro efficacy of thyme essential oil at sub-inhibitory concentrations (25%, 50%, and 75%) on the gene expression of *S. enteritidis* virulence factors, but also its effect on bacterial growth. The results showed that, after 18–72 h of incubation, the EO led to a reduction in bacterial density ranging from 2 to 4 log_10_ CFU/mL compared to the control. The most inhibitory effect was observed at the 75% concentration (1.25 mg/mL), which proved to be the most effective [47]. Yilmaz et al. [48] evaluated the antimicrobial activity of both extracts and EOs of zahter and bay leaf against *S. typhimurium*. The results showed that the bay leaf EO exhibited the strongest inhibitory effect compared to the other treatments [48]. In another study, the effectiveness of *Satureja hortensis* essential oil, whose main components included thymol (41.28%), γ-terpinene (37.63%), p-cymene (12.2%) and α-terpinene (3.52%) [49], was evaluated for its ability to eliminate biofilm and inhibit the growth of *Salmonella*. The results showed an inhibition zone of 38 ± 4 mm and demonstrated that biofilm formation was significantly reduced even at sub-inhibitory concentrations [49]. Table 2 lists the main EOs with efficacy in eliminating *Salmonella*, demonstrated through in vitro tests.

A recent study conducted on three of the most relevant pathogens in poultry production, including *S. typhimurium*, tested various EOs. The ones showing strong antimicrobial activity were *Origanum vulgare*, *Cinnamomum zeylanicum* and modified GR-OIL (a proprietary solution under patent development, containing nine EOs: *Eucalyptus globulus*, *Satureja hortensis*, *Citrus aurantium var. dulcis*, *Thymus vulgaris*, *Melaleuca alternifolia*, *Citrus limon*, *Lavandula* × *hybrida*, *Melaleuca leucadendron*, *and Thymus capitatus*) [51]. In particular, *Origanum vulgare* was found to be the most effective in inhibiting *S. typhimurium* [51]. Few studies have used EOs as biocides in poultry farming. Among these, Galgano et al. [45] examined the effects of thyme EO at various concentrations (from 1.2% to 5%) on the reduction in *S. derby* in experimentally contaminated poultry litter. The results indicated that the most effective antibacterial action was observed at the lowest concentration, showing a growth reduction of 77.8% compared to the positive control for *Salmonella*. These findings suggest that thyme EO could be used as a natural and effective approach for treating poultry litter [40]. Thyme was also studied by Witkowska et al. [53], who investigated the effectiveness of its nebulization, along with that of peppermint (*Mentha piperita*), in reducing bacterial contamination in a broiler farm. Other studies conducted on *S. senftenberg* isolates from poultry farms compared the effects of lingonberry extract (*Vaccinium vitis-idaea*) with those of low concentrations of formaldehyde [54]. The results showed that lingonberry extract was not yet as effective as formaldehyde. This suggests that although natural products, such as lingonberry extract, possess antibacterial potential, they are not yet as effective as traditional chemical disinfectants. However, replacing chemical agents with natural products remains a promising area of research, particularly in light of European restrictions on the use of formaldehyde in farming [54].

From the analysis of the studies, thymol, carvacrol and cinnamaldehyde emerge as highly effective natural agents against *Salmonella*, offering a sustainable alternative to traditional antimicrobials.

#### 5.4.3. Positive Biofilm

In recent years, the concept of “positive biofilm” has emerged as a potential antibacterial strategy. This refers to beneficial bacterial communities capable of forming a protective barrier that prevents the settlement of pathogens [20]. In C&D protocols, the term biofilm is traditionally associated with a negative connotation, as its natural resistance to antimicrobials makes its removal from surfaces challenging [20]. However, after C&D protocols and before the animals enter the facility, beneficial bacteria can be sprayed onto surfaces and building materials to colonise the “empty” biotope. This approach aims to prevent the establishment of undesirable microorganisms and limit their proliferation through competition for space and nutrients [20]. Currently, the products available on the market mainly consist of mixtures of lactic acid bacteria (LAB) or probiotics [20]. Recently, products based on live bacteria have been developed for application on surfaces and bedding in poultry farms. For example, ManurePRO is composed of bacteria such as *Bacillus velezensis*, *Pediococcus acidilactici*, and *Pediococcus pentosaceus* [77]. Similarly, LalfilmPRO contains LAB, *Bacillus subtilis*, *Bacillus amyloliquefaciens*, *Bacillus pumilus* and *Pediococcus* spp. Both products have been approved for use in organic poultry farming [55].

Maes et al. [56] proposed *Pseudomonas putida* as a biocontrol agent against *Salmonella* enterica subsp. enterica serovar Paratyphi B variant Java, also known as *S*. Java, in the drinking water systems of broiler chicken farms. The study examined whether the biofilm formation of different strains of *P. putida* could promote or inhibit the adhesion and biofilm formation of *S*. Java. To this end, an in vitro model was developed to simulate biofilm formation within the drinking water systems, closely replicating environmental conditions. The results demonstrated that the tested strains of *P. putida* could reduce the adhesion and biofilm formation of *S*. Java in the drinking water systems of broiler chicken farms [56].

**Figure 3:**
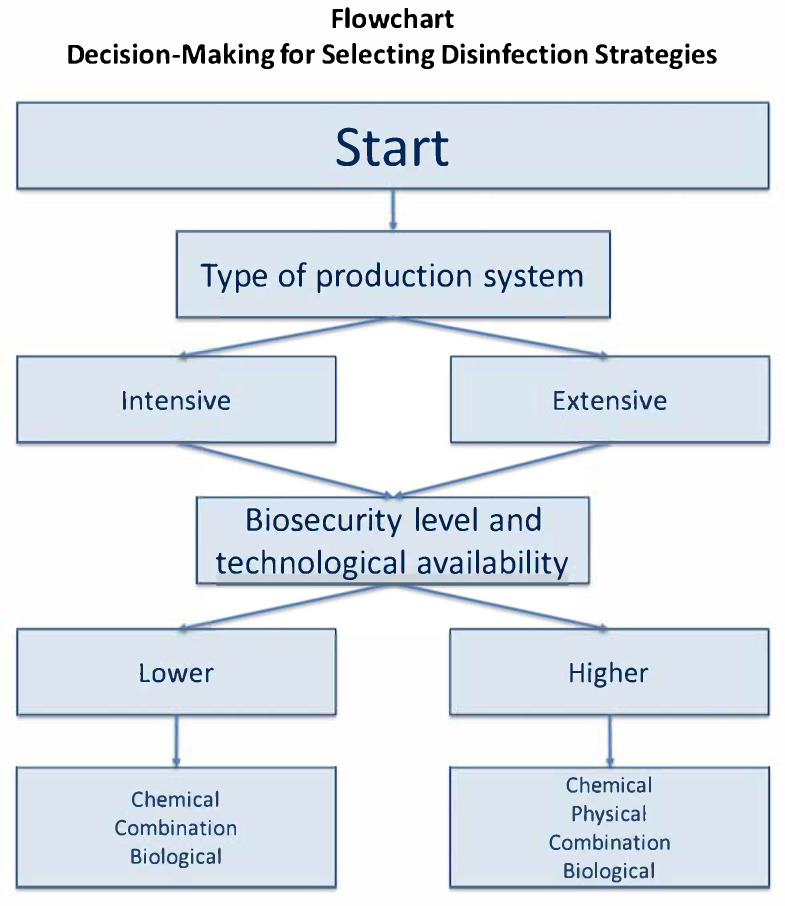
Decision-making flowchart for the selection of disinfection strategies in poultry farms.

How can we choose the best method? To this end, we propose a flowchart that can serve as a reference for selecting the most suitable method (Figure 3). The diagram illustrates the steps to consider when choosing C&D methods. The sequence starts with identification of the type of farm, followed by the level of biosecurity and the resources available on the farm, in order to select the most appropriate approach: chemical, physical, combined chemical-physical or biological.

## 6. European Legislation in C&D

The research and development of disinfectants represents a promising field worldwide [67]. Currently, there is strong pressure from consumers and legislators to reduce chemical disinfectant inputs in livestock farming to limit environmental impact and improve animal welfare and human health [8]. In this regard, Regulation (EC) No. 1907/2006 [78], concerning the registration, evaluation, authorisation and restriction of chemical substances, provides a framework for substances produced, imported, used and sold within the European Union (EU). In 2020, this regulation was updated as part of the Green Deal to promote strategies for more sustainable chemical use. This new strategy establishes a hierarchy of actions that encourage innovation, produce safe and sustainable chemicals, strengthen human health and environmental protection, and gradually reduce the use of toxic substances. Instead, Regulation (EU) No. 528/2012 [79] regulates the placing on the market and use of new biocides molecules, harmonising rules regarding the sale and use of these products to ensure a high level of human, animal and environmental health protection.

Regarding the other aforementioned methodologies, there is currently no specific legislation at the European level. For instance, in the case of physical methods, research focuses on food decontamination using physical techniques, such as irradiation [80]. However, there is a directive (Directive EU 2013/35) that establishes fundamental safety standards for workers regarding exposure to electromagnetic fields [81].

For bacteriophages, research is underway involving their use to reduce the presence of zoonotic pathogens in poultry farms for therapeutic purposes. Bacteriophage-based products can be bio-cleaning agents in hatcheries, farms, transport cages, poultry processing plants and food-contact surfaces [7]. However, there are currently no specific regulations governing their use [7,82,83]. Currently, phages used for therapeutic purposes are not considered biocides under Regulation (EU) No. 528/2012 [79]. Moreover, bacteriophages do not meet the quality requirements for substances defined by Article 3 of the same regulation, as only chemicals and their compounds are classified as such [82]. The lack of regulatory clarity slows down the development of commercially available bacteriophage-based products in Europe, although countries such as Poland, Russia and Georgia have been using them to treat infections since their discovery [7]. At present, the global market for bacteriophage-based preparations is underdeveloped but promising, with the United States leading research on phage-containing products. According to the latest report from Credence Research, “Bacteriophage Market Growth, Future, Competitive Analysis, 2018–2026,” the global bacteriophage market was estimated to be worth $567 million in 2017, with an average annual growth rate forecast of 3.9% [67]. Issabekov et al. [67] have developed a new broad-spectrum disinfectant, “Polyphage,” which is currently the most cost-effective compared to other “eco-friendly” disinfectants on the market, such as Medidezenzo or Miroseptik. The United States based company OmniLytics Inc. has recently launched a phage-based surface disinfectant against *Salmonella*.

A legislative gap exists regarding EOs, regardless of the type of farming considered. According to the official Pharmacopoeias, EOs are considered complex fragrant products obtained through steam distillation or hydrodistillation, or through dry distillation of a plant or its parts, or through appropriate cold mechanical processes. Meanwhile, the International Organisation for Standardisation (ISO) has established both conceptual criteria and quality criteria, which serve as global guidelines for essential oils [51]. Currently, *Origanum vulgare* is authorised as a feed additive, as listed in the EU Feed Additives Register according to Regulation (EC) No. 1831/2003 [84], and as a flavouring additive for all animal feeds under Regulation (EC) No. 1334/2008 [51,71,85]. Instead, regarding the use of positive bacteria, reference is made to Regulation (EC) No. 528/2012 [79] in Europe.

The lack of specific guidelines for these products represents a significant limitation for those wishing to adopt ecological solutions on their farms.

## 7. Biosecurity: The Role of the External Environment

Due to the ubiquitous nature of *Salmonella* in poultry, initial contact with the bacterium, if present in the environment, is inevitable. Consequently, control measures are implemented to protect the animals from direct contact with the pathogen [12]. C&D is an internal biosecurity measure within the farm, essential for eliminating pathogens before the start of a new production cycle, with particular attention to critical points to be cleaned, such as cracks, equipment and similar areas, where bacteria can hide and promote contamination [17,20]. However, preventing the introduction of pathogens into the system is equally important. This is achieved through external biosecurity protocols aimed at minimising the risk of pathogen entry into the farm [2,11,86]. In order to better illustrate the role of external biosecurity, Table 3 was expanded to indicate not only the main guidelines but also the specific external challenges they address, as well as how these measures contribute to reducing the risk of *Salmonella* introduction into poultry farms [87].

Humans and animals present on the farm (such as cats, dogs, insects and rodents) can be carriers of contamination and may be responsible for its transmission [20]. For example, good hygiene practices such as wearing shoe covers, hand washing, showering and proper waste management can help limit the spread of pathogens [20]. In the field of scientific research, the most common errors in biosecurity measures adopted to prevent the spread of *Salmonella* in poultry farms have been thoroughly studied. In a study conducted by Gosling et al. [88], the adoption of biosecurity measures in layer farms was assessed following an inspection visit. It was found that 83% of the farms tested (approximately 50/60) were affiliated with a company, which already entailed strict biosecurity measures. Nevertheless, 55% (29/53) received recommendations to improve boot hygiene, mainly through the use of more appropriate disinfectants and 14% (23/61) for hand hygiene. This result was somewhat predictable, as most of the farms studied used cages, where poultry generally has minimal contact with personnel [88]. In a similar study conducted in Ethiopia, it was observed that farm personnel generally did not adequately implement biosecurity measures. Abdi et al. [89] reported that 33.3% (3/9) of the personnel did not sanitise their hands after handling animals and boots were not disinfected before entering the farm. Furthermore, unrestricted access for unauthorised visitors posed an additional risk factor for the spread of *Salmonella* [89].

Marin et al. [90], in a previous study conducted in Spain on broiler farms, highlighted that farmers’ boots represent a high risk for *Salmonella* contamination, with a prevalence of 19.7% (12/61). Similarly, a study conducted in Nigeria by Jibril et al. [91] revealed that among 65 farms tested, 21 had no fencing, with a *Salmonella* positivity prevalence of 32.3% (7/21). Additionally, 36.9% of the farms (9 out of 24) did not disinfect boots, and the same percentage managed waste within the facilities. According to Akalu et al. [92], farms that managed waste disposal inside the farm had a higher percentage of positive *Salmonella* samples (18.2%, 6/33) compared to those that disposed of waste outside the farm (60%, 15/25). A similar result was observed regarding the lack of rodent control and poor hygiene practices by the staff [92]. Wales et al. [93], in a 12-month longitudinal study on environmental contamination in layer farms, reported a prevalence of 38.6% (34/88) of environmental vectors testing positive for *Salmonella*. During the summer, the presence of vectors increased significantly, contributing to a higher prevalence of the bacteria, especially in outdoor farms compared to indoor ones [93].

Finally, an interesting study conducted by Rabie et al. [94] investigated the anti-*Salmonella* activity of boot baths used in poultry farms. In general, they found that most of the chemical disinfectants used did not have sufficient bactericidal activity. However, the study included many variables, and it was observed that the presence of excessive organic matter, inadequate preparation of the disinfectants and infrequent replacement of the baths significantly impacted the effectiveness of the disinfectant [94]. Moreover, studies conducted in France and Spain revealed that, even after cleaning poultry houses, more than 50% remained *Salmonella*-positive, highlighting the importance of proper C&D procedures on poultry farms [90,95].

In 2014, the first biosecurity scoring system for the poultry sector, Biocheck.UGent™, was developed; this was specifically designed for each production system. This method is based on the priority principle, objectively classifying biosecurity measures according to their relevance in disease transmission, regardless of the specific pathogen involved. The system helps identify the strengths and weaknesses of the practices adopted by farmers, providing a basis for recommendations to improve biosecurity [96]. The study by Amalraj et al. [96] on the application of the Biocheck.UGent™ scoring system in poultry farms in Belgium, Poland, France, the Netherlands and Spain found lower scores for external biosecurity measures than for internal ones. However, it was observed that this difference in scores is because some aspects of external biosecurity are not always under the direct control of farmers. A similar biosecurity system was created and tested in farms in the Netherlands, Greece and Cyprus, and is known as BEAT [97].

## 8. Conclusions

By analysing a wide range of scientific studies, this review provides a comprehensive overview of the disinfection strategies in the poultry sector, highlighting both the challenges and opportunities in controlling *Salmonella* and other zoonotic pathogens.

Controlling *Salmonella* in poultry farms remains an ongoing challenge, further complicated by the emergence of serotypes resistant to traditional disinfectants. The prolonged use of chemical disinfectants has raised concerns not only regarding environmental impact and food safety, but also regarding the risk of selecting more resilient bacterial strains that are harder to eliminate through conventional C&D protocols. Throughout this review, the efficacy of the alternatives currently most valued has been assessed, particularly the combination of chemical–physical methods and biological approaches such as bacteriophages or EOs. These approaches have shown potential in lowering microbial loads on surfaces and litter in poultry environments. Among the alternatives considered, chemical methods remain the most widely used in real production contexts due to their proven efficacy and cost-effectiveness. However, bacteriophages are emerging as one of the most promising candidates, as they are easy to apply, economically advantageous, and safe for both humans and animals. As previously noted, the high specificity of bacteriophages toward their target bacteria ensures that they do not disrupt the surrounding environmental ecosystem and do not promote the emergence of resistance, thanks to the constant coevolution between phages and bacteria. At the same time, essential oils are attracting increasing interest as a natural and sustainable option: they combine broad-spectrum antimicrobial activity with a favourable safety profile and meet the growing consumer demand for residue-free solutions. Their large-scale application, however, remains limited, mainly due to variability in composition and stability issues (e.g., photosensitivity). Despite their potential, significant research gaps remain for both bacteriophages and essential oils, including the need to evaluate their long-term efficacy, performance under the high organic load typical of poultry farms and interactions with traditional C&D protocols (e.g., detergents), as well as regulatory aspects that are not yet fully defined.

The studies reviewed offer valid alternatives to current C&D protocols used in poultry farms. Most of the studies analysed are based on the synergistic effect of at least two methodologies to maximise the antimicrobial effect, even on complex surfaces that are difficult to sanitise. Although some results are based on in vitro trials, future research should evaluate their applicability in field conditions. Overall, the evidence suggests that there is no single “universal” solution for controlling *Salmonella*, but rather a range of options that should be evaluated and adapted based on the specific characteristics of each farm. To translate this knowledge into practice, we proposed a prioritisation framework to guide decision-making: (i) identify the type of production system (intensive vs. extensive); (ii) evaluate the baseline biosecurity level; and (iii) consider the technological availability on the farm. This framework aims to help farmers and veterinarians choose between the alternatives proposed.

Moreover, it is essential to strike a balance between protocol efficacy and economic sustainability, especially in the light of increasing societal demand for products from sustainable farms that respect animal welfare through extensive farming practices. In this context, strengthening biosecurity measures, particularly external ones, is crucial to limit the introduction and spread of *Salmonella*.

## Figures and Tables

**Table 1:** Summary of new methodologies to control *Salmonella* in poultry farms.

Type of Methods	Methods	Surfaces	Trials	Bacterial Strains	European Standards on Disinfectant Efficacy (EN1656 ^a^/EN14349 ^b^; Reduction log_10_)	Reference
**Chemical**	N-dodecyl-2-(pyridin-1-ium) acetamide chloride with chlorhexidine acetate and glutaraldehyde	Stainless steel and Poultry farm environment	in vitro: Antimicrobial activity assay in vivo: Toxicity tests on mice and chickens; Poultry farms disinfection test	*P. aeruginosa*, *S. aureus*, *E. coli*, *B. subtilis*, *E. hirae*, *P. vulgaris*, *C. albicans*	$\bar{x}$ 4.7 log_10_ (UFC/mL)	[11]
	Silver nanoparticles and Ca(OCl)_2_	Solid and liquid poultry waste	in vitro: Antimicrobial activity assay	*E. coli*, *Salmonella* spp., *K. pneumoniae* and *L. monocytogenes*	- *	[29]
	Silver nanoparticles	Polyethylene	in vitro: Antibiofilm activity assay	*S. enteritidis*	3.9 log_10_ (CFU/cm^2^)	[30]
**Physical**	UV ^1^-LED ^2^	Stainless steel and high-density polyethylene surfaces	in vitro: Antimicrobial activity assay	Cocktail of five *Salmonella* strains	$\bar{x}$ 3.4 log_10_ (CFU/cm^2^)	[31]
	PHI ^3^	Poultry litter	in vitro: Antimicrobial, antiviral, and antifungal activity assay	*E. coli*, *S. aureus*, *S. enterica* serovar Abony	~0.7 log_10_ (CFU/g)	[32]
	IR ^4^	Poultry litter (rice husks)	in vitro: Antimicrobial activity assay	*S. typhimurium*	4.0 log_10_ (CFU/g)	[33]
	Pressurised steam with forced hot air	Fibreglass and plastic floors	in vitro: Antimicrobial activity assay	*S. infantis* and *Enterobacteriaceae*	$\bar{x}$ 3.4 log_10_ (CFU/cm^2^)	[34]
**Combination Chemical/Physical/Biological**	SAEW ^5^ with UV ^1^	Plastic, stainless steel, glass and tyres	in vitro: Antimicrobial activity assay	*S. enteritidis*	$\bar{x}$ 4.3 and 6.1 log_10_ (CFU/cm^2^)	[35]
	PAA ^6^ or LAB ^7^ with UV-C	Stainless steel, silicone rubber and ultra-high molecular weight polyethylene.	in vitro: Antibiofilm activity assay	*S. enteritidis*	$\bar{x}$ 4.8 log_10_ (CFU/cm^2^)	[19]
	Surfactants and ClO_2_ with heat	Ceramic and stainless steel	in vitro: Antibiofilm activity assay	*S. enterica*	- *	[36]
	PAA ^6^ with heat	- *	in vitro: Antimicrobial activity assay	*S. enteritidis*, *S. derby*, *S. typhimurium* and *S. agona*	$\bar{x}$ ~3.25 log_10_ (CFU/mL)	[37]
	PAW ^8^	Stainless steel, PVC ^9^, wood and concrete	in vitro: Antimicrobial activity assay	*Salmonella* spp.	2.2 log _10_ (CFU)	[38]
	ECAW ^10^	Stainless steel and polyethylene	in vitro: Antibiofilm activity assay	*S. heidelberg*	$\bar{x}$ 2.6 log_10_ (CFU/cm^2^)	[39]
	IoT ^11^ robot system (combination UV light with ozone)	Simulation of poultry farm surface (including litter)	in vivo: Poultry farms decontamination test	*Enterobacteriaceae*	0.2 log_10_ (CFU/mL)	[40]
**Biological**	Bacteriophages	Poultry Farm environment	in vivo: Antimicrobial activity assay in 10 commercial poultry farms	*S. infantis*	$\bar{x}$ 2.3 log_10_ (CFU/mL)	[3]
		Stainless steel and poultry drinkers	in vitro: Antibiofilm activity assay	*S. enteritidis*	- *	[41]
		Stainless steel	in vitro: Antimicrobial activity assay	*S. enteritidis*, *S. typhimurium*, *S. infantis*, *S. paratyphi B.* and *S. indiana*	*x* > 2.0 log_10_ (CFU/cm^2^)	[42]
		Poultry litter	in vitro: Antimicrobial activity assay	*S. enteritidis*	3.0 log_10_ (CFU/g)	[43]
		Metal	in vitro: Antibiofilm activity assay	*S. enteritidis*	0.9 log_10_ (CFU/mL)	[44]
	EO ^12^	- *	in vitro: Antimicrobial and Antibiofilm activity assay	*S. derby*; *S. enteritidis*; *S. typhimurium*	- *	[45,46,47,48,49,50,51]
	Plant extraction	- *	in vitro: Antimicrobial and Antibiofilm activity assay	*Salmonella* spp.	- *	[52]
	Nebulising peppermint and thyme EO	Poultry Farm environment	in vivo: Antimicrobial activity assay	*Enterobacteriaceae*	$\bar{x}$ ~0.6 log_10_ (CFU/mL)	[53]
	Formaldehyde and blueberry extract	- *	in vitro: Antibiofilm activity assay	*S. senftenberg* and *E. coli*	- *	[54]
	LAB	Polystyrene plates, wood shavings and soil samples	in vitro: Antibiofilm activity assay	*S. gallinarum*, *S. heidelberg*, *C. jejuni* and methicillin resistant *S. aureus*	*x* 7.3 log_10_ (CFU/mL)	[55]
	*Pseudomonas putida*	Drinking water	in vitro: Antibiofilm activity assay	*S. java*	- *	[56]

^1^ UV: Ultraviolet light; ^2^ LED: Light-emitting diodes; ^3^ PHI: Photohydroionization; ^4^ IR: Infrared radiation; ^5^ SAEW: Slightly acidic electrolysed water; ^6^ PAA: Peroxyacetic acid; ^7^ LAB: Latic acid bacteria; ^8^ PAW: Plasma-activated water; ^9^ PVC: Polyvinyl chloride; ^10^ ECAW: Electrochemically activated water; ^11^ IoT: Internet of Things; ^12^ EO: Essential oil; * (-) Not Available; * refer to Table 2; ^a^ EN1656: reduction (log_10_) ≥ 5; ^b^ EN14349: reduction (log_10_) ≥ 4.

**Table 2:** Main essential oils with proven efficacy in eliminating *Salmonella*.

Scientific Name	Essential Oil/ Main Component	*Salmonella* Strains	Trials	MIC/log_10_	References
*Laurus nobilis*	Bay leaf extract and oil	*S. typhimurium*	in vitro: Antimicrobial activity assay	64 mg/mL and 0.2 µL/mL	[48]
*Satureja hortensis*	Thymol	*Salmonella* spp.		0.31 a 0.62 μL/mL	[49]
*Thymus vulgaris* L., *Origanum vulgare*	Thymol and Carvacrol	*S. derby*	in vitro: Antimicrobial activity assay on poultry litter	*x* 5.79 log_10_ (CFU/g)	[45]
		*S. enteritidis*	in vitro: Antimicrobial activity assay	128 μg/mL and 256 μg/mL	[46]
		*S. enteritidis*		from 2 to 4 log_10_ (CFU/mL)	[47]
		*S. typhimurium*, *S. infantis*		20 μL/mL	[71]
		*S. typhimurium*		from 0.06 to 0.38 (% *v*/*v*)	[51]
		*Salmonella* spp.		from 320 to 640 μg/mL	[72]
*Thymbra spicata*	Zahter extract and oil	*S. typhimurium*	in vitro: Antimicrobial activity assay	0.2 µL/mL	[48]
*Cinnamomum zeylanicum*	Cinnamaldehyde	*S. enteritidis*	in vitro: Antimicrobial activity assay	128 μg/mL	[46]
		*S. enteritidis*		0.06% and 0.31% (% *v*/*v*)	[51]
		*S. typhimurium*		from 1.26 to 0.63 mg/mL	[50]
*Vaccinium vitis-idaea*	Lingonberry extract	*S. senftenberg*	in vitro: Antimicrobial activity assay	0.02 mg/mL	[54]
*Syzygium aromaticum*	Eugenol	*S. typhimurium*	in vitro: Antimicrobial activity assay	from 2.637 to 0.164 mg/mL	[50]
*Nigella sativa*	Black seed extract and oil	*Salmonella enterica*	in vitro: Antimicrobial activity assay	≥562.5 and ≥1000.0 μg/mL	[73]
*Pimenta officinalis*	Pimenta leaf	*S. heidelberg*	in vitro: Antimicrobial activity assay on chicken skin	>2 log_10_ CFU/in^2^	[74]
*Lippia graveolens*	Carvacrol and Thymol	*S. typhimurium*	in vitro: Antimicrobial and Antibiofilm activity assay on stainless steel	0.250 mg/mL^−1^ and 0.150 mg/mL^−1^	[75]
*Litsea cubeba*	Litsea	monophasic *S. typhimurium*	in vitro: Antimicrobial activity assay	0.4 mg/mL	[76]
*Allium sativum*	Garlic	*S. typhimurium*	in vitro: Antimicrobial activity assay	- *	[69]

* (-) Not Available.

**Table 3:** Main biosecurity guidelines can be applied to minimise the risk of *Salmonella* transmission from external to internal farm environment [87].

Guideline	External Challenge Addressed	How It Assists
Restrict access to visitors (ensure that visitors are provided with clean clothing and disinfected boots before entry)	Risk of pathogen introduction via humans	Minimises direct *Salmonella* entry from contaminated footwear/clothing
Regular C&D of the poultry farm	Risk of *Salmonella* transmission to new animal batches	Limits the transmission of *Salmonella* from existing animals to newly introduced batches in the farm.
Workers must wear clean clothing and ensure their boots are disinfected before entering the farm	Workers’ footwear/clothing contaminated by the external environment	Reduces the transfer of *Salmonella* into animal housing
Proper waste management	Waste inside the farm may attract pests and harbour pathogens	Limits the persistence of *Salmonella* and prevents indirect transmission
Vehicle access must be restricted and must be C&D before entering the farm.	Vehicles can carry contaminated litter, feed, or equipment	Reduces cross-contamination between farms
Rodent/insect control	Rodents and insects act as *Salmonella* reservoirs and vectors	Prevents the spread of bacteria from the surrounding environment into poultry houses

## Data Availability

Not applicable.

## Abbreviations

The following abbreviations are used in this manuscript:

AgNP	Silver nanoparticles
AuNP	Gold nanoparticles
C&D	Cleaning and Disinfection
CFU	Colony Forming Unit
EC	European Community
ECAW	Electrochemically Activated Water
EO	Essential Oil
EU	European Union
FDA	Food and Drug Administration
G−	Gram-negative
G+	Gram-positive
IoT	Internet of Things
IR	Infrared radiation
ISO	International Organisation for Standardisation
LAB	Latic Acid Bacteria
LED	Light-emitting diodes
MIC	Minimum Inhibitory Concentrations
PAA	Peroxyacetic Acid
PAW	Plasma-Activated Water
PFU	Plaque Forming Unit
PVC	Polyvinyl chloride
QAC	Quaternary Ammonium Compounds
SAEW	Slightly Acidic Electrolysed Water
UV	Ultraviolet Light

## References

[1] Montoro-Dasi L., Lorenzo-Rebenaque L., Marco-Fuertes A., Vega S., Marin C. (2023). Holistic Strategies to Control *Salmonella* Infantis: An Emerging Challenge in the European Broiler Sector. Microorganisms.

[2] Gentile N., Carrasquer F., Marco-Fuertes A., Marin C. (2024). Backyard Poultry: Exploring Non-Intensive Production Systems. Poult. Sci..

[3] Sevilla-Navarro S., Torres-Boncompte J., Garcia-Llorens J., Bernabéu-Gimeno M., Domingo-Calap P., Catalá-Gregori P. (2024). Fighting *Salmonella* Infantis: Bacteriophage-Driven Cleaning and Disinfection Strategies for Broiler Farms. Front. Microbiol..

[4] EFSA (2024). The European Union One Health 2023 Zoonoses Report.

[5] Kapoor R., Azad S., Mukherjee A., Dhar S. (2018). Reiter’s Disease in a 8-Year-Old Boy. Indian J. Paediatr. Dermatol..

[6] Liu X., Jiang Z., Liu Z., Li D., Liu Z., Dong X., Yan S., Zhu L., Cui D., Chen L. (2023). Biofilm-Forming Ability of *Salmonella enterica* Strains of Different Serotypes Isolated from Multiple Sources in China. Microb. Pathog..

[7] Jordá J., Lorenzo-Rebenaque L., Montoro-Dasi L., Marco-Fuertes A., Vega S., Marin C. (2023). Phage-Based Biosanitation Strategies for Minimizing Persistent *Salmonella* and *Campylobacter* Bacteria in Poultry. Animals.

[8] Flemming H.-C., Wingender J., Szewzyk U., Steinberg P., Rice S.A., Kjelleberg S. (2016). Biofilms: An Emergent Form of Bacterial Life. Nat. Rev. Microbiol..

[9] Obe T., Kiess A.S., Nannapaneni R. (2024). Antimicrobial Tolerance in *Salmonella*: Contributions to Survival and Persistence in Processing Environments. Animals.

[10] Cadena M., Kelman T., Marco M.L., Pitesky M. (2019). Understanding Antimicrobial Resistance (AMR) Profiles of *Salmonella* Biofilm and Planktonic Bacteria Challenged with Disinfectants Commonly Used During Poultry Processing. Foods.

[11] Chen N., Qin P., Liu Y., Yang Y., Wen H., Jia L., Li J., Zhu Z. (2021). Influence of New Compound Disinfectant From N-Dodecyl-2-(Piridin-1-Ium)Acetamide Chloride on Pathogenic Microorganisms in Poultry Houses. Front. Microbiol..

[12] Obe T., Boltz T., Kogut M., Ricke S.C., Brooks L.A., Macklin K., Peterson A. (2023). Controlling *Salmonella*: Strategies for Feed, the Farm, and the Processing Plant. Poult. Sci..

[13] Cawthraw S., Wales A., Guzinski J., Trew J., Ring I., Huby T., Hussaini A., Petrovska L., Martelli F. (2025). *Salmonella* Infantis Outbreak on Six Broiler Units in Great Britain: Investigation, Epidemiology, and Control. J. Appl. Microbiol..

[14] Münster P., Pöppel L., Antakli A., Müller-Doblies D., Radko D., Kemper N. (2023). The Detection of *Salmonella* Enteritidis on German Layer Farms after Cleaning and Disinfection. Animals.

[15] Zeng H., De Reu K., Gabriël S., Mattheus W., De Zutter L., Rasschaert G. (2021). *Salmonella* Prevalence and Persistence in Industrialized Poultry Slaughterhouses. Poult. Sci..

[16] Obe T., Nannapaneni R., Schilling W., Zhang L., McDaniel C., Kiess A. (2020). Prevalence of *Salmonella enterica* on Poultry Processing Equipment after Completion of Sanitization Procedures. Poult. Sci..

[17] Castañeda-Gulla K., Sattlegger E., Mutukumira A.N. (2020). Persistent Contamination of Salmonell, Campylobacter, *Escherichia coli*, and *Staphylococcus aureus* at a Broiler Farm in New Zealand. Can. J. Microbiol..

[18] Luyckx K.Y., Van Weyenberg S., Dewulf J., Herman L., Zoons J., Vervaet E., Heyndrickx M., De Reu K. (2015). On-Farm Comparisons of Different Cleaning Protocols in Broiler Houses. Poult. Sci..

[19] Byun K.-H., Na K.W., Ashrafudoulla M., Choi M.W., Han S.H., Kang I., Park S.H., Ha S.-D. (2022). Combination Treatment of Peroxyacetic Acid or Lactic Acid with UV-C to Control *Salmonella* Enteritidis Biofilms on Food Contact Surface and Chicken Skin. Food Microbiol..

[20] Guéneau V., Plateau-Gonthier J., Arnaud L., Piard J.-C., Castex M., Briandet R. (2022). Positive Biofilms to Guide Surface Microbial Ecology in Livestock Buildings. Biofilm.

[21] Aljuwayd M., Malli I.A., Olson E.G., Ricke S.C., Rothrock M.J., Kwon Y.M. (2025). Disinfectants and One Health Review: The Role of Reactive Oxygen Species in the Bactericidal Activity of Chlorine against *Salmonella*. One Health.

[22] Krüger G.I., Urbina F., Pardo-Esté C., Salinas V., Álvarez J., Avilés N., Oviedo A., Kusch C., Pavez V., Vernal R. (2025). Resilient by Design: Environmental Stress Promotes Biofilm Formation and Multi-Resistance in Poultry-Associated *Salmonella*. Microorganisms.

[23] Jang Y., Lee K., Yun S., Lee M., Song J., Chang B., Choe N. (2017). Efficacy Evaluation of Commercial Disinfectants by Using *Salmonella enterica* Serovar Typhimurium as a Test Organism. J. Vet. Sci..

[24] Megahed A., Aldridge B., Lowe J. (2019). Comparative Study on the Efficacy of Sodium Hypochlorite, Aqueous Ozone, and Peracetic Acid in the Elimination of *Salmonella* from Cattle Manure Contaminated Various Surfaces Supported by Bayesian Analysis. PLoS ONE.

[25] Corcoran M., Morris D., De Lappe N., O’Connor J., Lalor P., Dockery P., Cormican M. (2014). Commonly Used Disinfectants Fail To Eradicate *Salmonella enterica* Biofilms from Food Contact Surface Materials. Appl. Environ. Microbiol..

[26] Fredell D.L., Cords B.B., Givins B.J. (1985). Effect of pH and Water Hardness on the Sanitizing Activity of Five Commercial Lodophors. J. Food Prot..

[27] Kim H.-J., Tango C.N., Chelliah R., Oh D.-H. (2019). Sanitization Efficacy of Slightly Acidic Electrolyzed Water against Pure Cultures of *Escherichia coli*, *Salmonella enterica*, *Typhimurium*, *Staphylococcus aureus* and *Bacillus cereus* Spores, in Comparison with Different Water Hardness. Sci. Rep..

[28] Kroft B., Leone C., Wang J., Kataria J., Sidhu G., Vaddu S., Bhumanapalli S., Berry J., Thippareddi H., Singh M. (2024). Influence of Peroxyacetic Acid Concentration, Temperature, pH, and Treatment Time on Antimicrobial Efficacy against *Salmonella* on Chicken Wings. Poult. Sci..

[29] Mohammed A.N. (2022). An Alternative Approach for Controlling Bacterial Pathogens in Liquid and Solid Poultry Waste Using Calcium Hypochlorite Ca(OCl)_2_ Disinfectant-Based Silver Nanoparticles. Sci. Rep..

[30] Dias De Emery B., Zottis Chitolina G., Qadir M.I., Quedi Furian T., Apellanis Borges K., De Souza Moraes H.L., Pippi Salle C.T., Pinheiro Do Nascimento V. (2023). Antimicrobial and Antibiofilm Activity of Silver Nanoparticles against *Salmonella* Enteritidis. Braz. J. Microbiol..

[31] Calle A., Fernandez M., Montoya B., Schmidt M., Thompson J. (2021). UV-C LED Irradiation Reduces *Salmonella* on Chicken and Food Contact Surfaces. Foods.

[32] Appel A., Zuffo J.P., Wolf J., Stahlhofer S.R., Lopes P.D., Correia B., Moreira F., Millezi A.F., Bianchi I., Oliveira Júnior J.M. (2021). Photohydroionisation for Disinfection of Poultry Litter. Br. Poult. Sci..

[33] Kaewthong J., Satienpaisan W., Krabuansang K., Artsri J., Chaisakul P., Chiangga S., Chattham N., Samipak S. (2023). Experiment and Simulation of Heat Treatment for Disinfection in the Chicken Farm Using IR Irradiation. J. Phys. Conf. Ser..

[34] Reina M., Urrutia A., Figueroa J.C., Riggs M.R., Macklin K.S., Buhr R.J., Price S.B., Bourassa D.V. (2024). Application of Pressurized Steam and Forced Hot Air for Cleaning Broiler Transport Container Flooring. Poult. Sci..

[35] Zang Y.T., Bing S., Li Y.J., Shu D.Q. (2019). Application of Slightly Acidic Electrolyzed Water and Ultraviolet Light for *Salmonella* Enteritidis Decontamination of Cell Suspensions and Surfaces of Artificially Inoculated Plastic Poultry Transport Coops and Other Facility Surfaces. Poult. Sci..

[36] Ohashi I., Kobayashi S., Tamamura-Andoh Y., Arai N., Takamatsu D. (2022). Disinfectant Resistance of *Salmonella* in in Vitro Contaminated Poultry House Models and Investigation of Efficient Disinfection Methods Using These Models. J. Vet. Med. Sci..

[37] Šovljanski O., Ranitović A., Tomić A., Ćetković N., Miljković A., Saveljić A., Cvetković D. (2023). Synergistic Strategies of Heat and Peroxyacetic Acid Disinfection Treatments for *Salmonella* Control. Pathogens.

[38] Měřínská T., Walker M., Keener K. (2025). Using Plasma-Activated Water for Decontamination of *Salmonella* spp. on Common Building Surfaces in Poultry Houses. Food Microbiol..

[39] Wilsmann D.E., Furian T.Q., Carvalho D., Chitolina G.Z., Lucca V., Emery B.D., Borges K.A., Martins A.C., Pontin K.P., Salle C.T.P. (2023). Antibiofilm Activity of Electrochemically Activated Water (ECAW) in the Control of *Salmonella* Heidelberg Biofilms on Industrial Surfaces. Braz. J. Microbiol..

[40] Kunz Cechinel A., Soares C.E., Pfleger S.G., De Oliveira L.L.G.A., Américo De Andrade E., Damo Bertoli C., De Rolt C.R., De Pieri E.R., Plentz P.D.M., Röning J. (2024). Mobile Robot + IoT: Project of Sustainable Technology for Sanitizing Broiler Poultry Litter. Sensors.

[41] Korzeniowski P., Śliwka P., Kuczkowski M., Mišić D., Milcarz A., Kuźmińska-Bajor M. (2022). Bacteriophage Cocktail Can Effectively Control *Salmonella* Biofilm in Poultry Housing. Front. Microbiol..

[42] Gvaladze T., Lehnherr H., Hertwig S. (2024). A Bacteriophage Cocktail Can Efficiently Reduce Five Important *Salmonella* Serotypes Both on Chicken Skin and Stainless Steel. Front. Microbiol..

[43] Rogovski P., Silva R.D., Cadamuro R.D., Souza E.B.D., Savi B.P., Viancelli A., Michelon W., Tápparo D.C., Treichel H., Rodríguez-Lazaro D. (2021). *Salmonella enterica* Serovar Enteritidis Control in Poultry Litter Mediated by Lytic Bacteriophage Isolated from Swine Manure. Int. J. Environ. Res. Public Health.

[44] Ge H., Lin C., Xu Y., Hu M., Xu Z., Geng S., Jiao X., Chen X. (2022). A Phage for the Controlling of *Salmonella* in Poultry and Reducing Biofilms. Vet. Microbiol..

[45] Galgano M., Pellegrini F., Fracchiolla G., Mrenoshki D., Zarea A.A.K., Bianco A., Del Sambro L., Capozzi L., Schiavone A., Saleh M.S. (2023). Pilot Study on the Action of Thymus Vulgaris Essential Oil in Treating the Most Common Bacterial Contaminants and *Salmonella enterica* Subsp. Enterica Serovar Derby in Poultry Litter. Antibiotics.

[46] Wang W., Li T., Chen J., Ye Y. (2023). Inhibition of *Salmonella* Enteritidis by Essential Oil Components and the Effect of Storage on the Quality of Chicken. Foods.

[47] Hassanzadeh M., Mirzaie S., Pirmahalle F.R., Yahyaraeyat R., Razmyar J. (2024). Effects of Thyme (*Thymus Vulgaris*) Essential Oil on Bacterial Growth and Expression of Some Virulence Genes in *Salmonella enterica* Serovar Enteritidis. Vet. Med. Sci..

[48] Yilmaz E.A., Yalçin H., Polat Z. (2024). Antimicrobial Effects of Laurel Extract, Laurel Essential Oil, Zahter Extract, and Zahter Essential Oil on Chicken Wings Contaminated with *Salmonella typhimurium*. Vet. Med. Sci..

[49] Haji Seyedtaghiya M., Nayeri Fasaei B., Peighambari S.M. (2021). Antimicrobial and Antibiofilm Effects of *Satureja hortensis* Essential Oil against *Escherichia coli* and *Salmonella* Isolated from Poultry. Iran. J. Microbiol..

[50] Ebani V.V., Nardoni S., Bertelloni F., Pollera C., Pistelli L., Mancianti F. (2021). In Vitro Antimicrobial Activity of Selected Essential Oils against Bacteria and Yeasts Isolated from the Genital Tract of Mares. Nat. Prod. Res..

[51] Mariotti M., Lombardini G., Rizzo S., Scarafile D., Modesto M., Truzzi E., Benvenuti S., Elmi A., Bertocchi M., Fiorentini L. (2022). Potential Applications of Essential Oils for Environmental Sanitization and Antimicrobial Treatment of Intensive Livestock Infections. Microorganisms.

[52] Olawuwo O.S., Famuyide I.M., McGaw L.J. (2022). Antibacterial and Antibiofilm Activity of Selected Medicinal Plant Leaf Extracts Against Pathogens Implicated in Poultry Diseases. Front. Vet. Sci..

[53] Witkowska D., Sowińska J. (2013). The Effectiveness of Peppermint and Thyme Essential Oil Mist in Reducing Bacterial Contamination in Broiler Houses. Poult. Sci..

[54] Choroszy-Król I., Futoma-Kołoch B., Kuźnik K., Wojnicz D., Tichaczek-Goska D., Frej-Mądrzak M., Jama-Kmiecik A., Sarowska J. (2023). Exposing *Salmonella* Senftenberg and *Escherichia coli* Strains Isolated from Poultry Farms to Formaldehyde and Lingonberry Extract at Low Concentrations. Int. J. Mol. Sci..

[55] Monteiro G., Rossi D., Valadares E., Peres P., Braz R., Notário F., Gomes M., Silva R., Carrijo K., Fonseca B. (2021). Lactic Bacterium and *Bacillus* sp. Biofilms Can Decrease the Viability of *Salmonella gallinarum*, *Salmonella* Heidelberg, *Campylobacter jejuni* and Methicillin Resistant *Staphylococcus aureus* on Different Substrates. Braz. J. Poult. Sci..

[56] Maes S., De Reu K., Van Weyenberg S., Lories B., Heyndrickx M., Steenackers H. (2020). Pseudomonas Putida as a Potential Biocontrol Agent against *Salmonella* Java Biofilm Formation in the Drinking Water System of Broiler Houses. BMC Microbiol..

[57] Crisan C.M., Mocan T., Manolea M., Lasca L.I., Tăbăran F.-A., Mocan L. (2021). Review on Silver Nanoparticles as a Novel Class of Antibacterial Solutions. Appl. Sci..

[58] Zhang X.-F., Liu Z.-G., Shen W., Gurunathan S. (2016). Silver Nanoparticles: Synthesis, Characterization, Properties, Applications, and Therapeutic Approaches. Int. J. Mol. Sci..

[59] Rose G.K., Thakur B., Soni R., Soni S.K. (2023). Biosynthesis of Silver Nanoparticles Using Nitrate Reductase from *Aspergillus terreus* N4 and Their Potential Use as a Non-Alcoholic Disinfectant. J. Biotechnol..

[60] Fadaka A., Aluko O., Awawu S., Theledi K. (2021). Green Synthesis of Gold Nanoparticles Using Pimenta Dioica Leaves Aqueous Extract and Their Application as Photocatalyst, Antioxidant, and Antibacterial Agents. J. Multidiscip. Appl. Nat. Sci..

[61] Gupta A., Pandey B.C., Yaseen M., Kushwaha R., Verma J., Manna P.P., Manhas R.K., Tiwari I., Kumari N. (2025). Biofabrication of Gold Nanoparticles (GNPs) Synthesized from *Dillenia indica* Leaves with Their Anticancer, Antibacterial, and Antioxidant Activities. bioRxiv.

[62] Amaral A.L., Aoki A., Andrade S.A. (2024). Could Light Be a Broad-Spectrum Antimicrobial?. Evid.-Based Dent..

[63] European Parliament and Council (2011). Regulation (EU) No 142/2011 of 25 February 2011 Implementing Regulation (EC) No 1069/2009 of the European Parliament and of the Council Laying down Health Rules as Regards Animal by-Products and Derived Products Not Intended for Human Consumption and Implementing Council Directive 97/78/EC as Regards Certain Samples and Items Exempt from Veterinary Checks at the Border under That Directive Text with EEA Relevance.

[64] Berrang M.E., Meinersmann R.J., Cox N.A., Adams E.S. (2020). Water Rinse and Flowing Steam to Kill Campylobacter on Broiler Transport Coop Flooring. Food Control.

[65] Hao X.X., Li B.M., Wang C.Y., Zhang Q., Cao W. (2013). Application of Slightly Acidic Electrolyzed Water for Inactivating Microbes in a Layer Breeding House. Poult. Sci..

[66] Ni L., Cao W., Zheng W.C., Zhang Q., Li B.M. (2015). Reduction of Microbial Contamination on the Surfaces of Layer Houses Using Slightly Acidic Electrolyzed Water. Poult. Sci..

[67] Issabekov S.S., Syrym N.S., Sambetbayev A.A., Alikhanov K.D., Yespembetov B.A. (2022). Prospects of Bacteriophage Collections in Disinfectant Applications. Vet. World.

[68] Gildea L., Ayariga J.A., Robertson B.K. (2022). Bacteriophages as Biocontrol Agents in Livestock Food Production. Microorganisms.

[69] Oliveira G.D.S., McManus C., Sousa H.A.D.F., Santos P.H.G.D.S., Dos Santos V.M. (2024). A Mini-Review of the Main Effects of Essential Oils from *Citrus aurantifolia*, *Ocimum basilicum*, and *Allium sativum* as Safe Antimicrobial Activity in Poultry. Animals.

[70] Wińska K., Mączka W., Łyczko J., Grabarczyk M., Czubaszek A., Szumny A. (2019). Essential Oils as Antimicrobial Agents—Myth or Real Alternative?. Molecules.

[71] Di Vito M., Cacaci M., Barbanti L., Martini C., Sanguinetti M., Benvenuti S., Tosi G., Fiorentini L., Scozzoli M., Bugli F. (2020). *Origanum vulgare* Essential Oil vs. a Commercial Mixture of Essential Oils: In Vitro Effectiveness on *Salmonella* spp. from Poultry and Swine Intensive Livestock. Antibiotics.

[72] Boskovic M., Djordjevic J., Ivanovic J., Janjic J., Zdravkovic N., Glisic M., Glamoclija N., Baltic B., Djordjevic V., Baltic M. (2017). Inhibition of *Salmonella* by Thyme Essential Oil and Its Effect on Microbiological and Sensory Properties of Minced Pork Meat Packaged under Vacuum and Modified Atmosphere. Int. J. Food Microbiol..

[73] Ashraf S., Anjum A.A., Ahmad A., Firyal S., Sana S., Latif A.A. (2018). In Vitro Activity of Nigella Sativa against Antibiotic Resistant *Salmonella enterica*. Environ. Toxicol. Pharmacol..

[74] Nair D.V.T., Kollanoor Johny A. (2017). Food Grade Pimenta Leaf Essential Oil Reduces the Attachment of *Salmonella enterica* Heidelberg (2011 Ground Turkey Outbreak Isolate) on to Turkey Skin. Front. Microbiol..

[75] Luna-Solorza J.M., Ayala-Zavala J.F., Cruz-Valenzuela M.R., González-Aguilar G.A., Bernal-Mercado A.T., Gutierrez-Pacheco M.M., Silva-Espinoza B.A. (2023). Oregano Essential Oil versus Conventional Disinfectants against *Salmonella typhimurium* and *Escherichia coli* O157:H7 Biofilms and Damage to Stainless-Steel Surfaces. Pathogens.

[76] Wang C., Chen X., Liu M., Tang X., Li Y., Zhan Y., Hao Z. (2025). Antibacterial Activity and Mechanism of *Litsea cubeba* Essential Oil Against *Salmonella typhimurium*. Plants.

[77] Lallemand Animal Nutrition Triple-action Bedding and Manure Solution: MANURE PRO. https://www.lallemandanimalnutrition.com/es/spain/productos/manure-pro/.

[78] European Parliament and Council (2006). Regulation (EC) No 1907/2006 of the European Parliament and of the Council of 18 December 2006 Concerning the Registration, Evaluation, Authorisation and Restriction of Chemicals (REACH).

[79] European Parliament and Council (2012). Regulation (EU) No 528/2012 of the European Parliament and of the Council of 22 May 2012 Concerning the Making Available on the Market and Use of Biocidal Products.

[80] EFSA Autorità Europea per la Sicurezza Alimentare L’EFSA Valuta la Sicurezza Degli Alimenti Irradiati. https://www.efsa.europa.eu/it/news/efsa-assesses-safety-food-irradiation.

[81] European Parliament and Council (2013). Directive 2013/35/EU of the European Parliament and of the Council of 26 June 2013 on the Minimum Health and Safety Requirements Regarding the Exposure of Workers to Risks Arising from Physical Agents (Electromagnetic Fields).

[82] Faltus T. (2024). The Medicinal Phage—Regulatory Roadmap for Phage Therapy under EU Pharmaceutical Legislation. Viruses.

[83] Naureen Z., Dautaj A., Anpilogov K., Camilleri G., Dhuli K., Tanzi B., Maltese P.E., Cristofoli F., De Antoni L., Beccari T. (2020). Bacteriophages Presence in Nature and Their Role in the Natural Selection of Bacterial Populations. Acta Bio Medica Atenei Parm..

[84] European Parliament and Council (2003). Regulation (EC) No 1831/2003 of the European Parliament and of the Council of 22 September 2003 on Additives for Use in Animal Nutrition.

[85] European Parliament and Council (2008). Regulation (EC) No 1334/2008 of the European Parliament and of the Council of 16 December 2008 on Flavourings and Certain Food Ingredients with Flavouring Properties for Use in and on Foods and Amending Council Regulation (EEC) No 1601/91, Regulations (EC) No 2232/96 and (EC) No 110/2008 and Directive 2000/13/EC.

[86] Wang J., Vaddu S., Bhumanapalli S., Mishra A., Applegate T., Singh M., Thippareddi H. (2023). A Systematic Review and Meta-Analysis of the Sources of *Salmonella* in Poultry Production (Pre-Harvest) and Their Relative Contributions to the Microbial Risk of Poultry Meat. Poult. Sci..

[87] Lamichhane B., Mawad A.M.M., Saleh M., Kelley W.G., Harrington P.J., Lovestad C.W., Amezcua J., Sarhan M.M., El Zowalaty M.E., Ramadan H. (2024). Salmonellosis: An Overview of Epidemiology, Pathogenesis, and Innovative Approaches to Mitigate the Antimicrobial Resistant Infections. Antibiotics.

[88] Gosling R.J., Martelli F., Wintrip A., Sayers A.R., Wheeler K., Davies R.H. (2014). Assessment of Producers’ Response to *Salmonella* Biosecurity Issues and Uptake of Advice on Laying Hen Farms in England and Wales. Br. Poult. Sci..

[89] Abdi R.D., Mengstie F., Beyi A.F., Beyene T., Waktole H., Mammo B., Ayana D., Abunna F. (2017). Determination of the Sources and Antimicrobial Resistance Patterns of *Salmonella* Isolated from the Poultry Industry in Southern Ethiopia. BMC Infect. Dis..

[90] Marin C., Balasch S., Vega S., Lainez M. (2011). Sources of *Salmonella* Contamination during Broiler Production in Eastern Spain. Prev. Vet. Med..

[91] Jibril A.H., Okeke I.N., Dalsgaard A., Kudirkiene E., Akinlabi O.C., Bello M.B., Olsen J.E. (2020). Prevalence and Risk Factors of *Salmonella* in Commercial Poultry Farms in Nigeria. PLoS ONE.

[92] Akalu A., Tadesse T., Alemayehu H., Medhin G., Woldeyohannes D., Eguale T. (2024). Prevalence and Antimicrobial Susceptibility Profile of *Salmonella* from Poultry Farms and In-Contact Humans and Associated Risk Factors in Addis Ababa, Ethiopia. Int. J. Microbiol..

[93] Wales A., Breslin M., Carter B., Sayers R., Davies R. (2007). A Longitudinal Study of Environmental *Salmonella* Contamination in Caged and Free-Range Layer Flocks. Avian Pathol..

[94] Rabie A.J., McLaren I.M., Breslin M.F., Sayers R., Davies R.H. (2015). Assessment of Anti-*Salmonella* Activity of Boot Dip Samples. Avian Pathol..

[95] Rose N., Beaudeau F., Drouin P., Toux J.Y., Rose V., Colin P. (2000). Risk Factors for *Salmonella* Persistence after Cleansing and Disinfection in French Broiler-Chicken Houses. Prev. Vet. Med..

[96] Amalraj A., Van Meirhaeghe H., Caekebeke N., Creve R., Dufay-Lefort A.-C., Rousset N., Spaans A., Devesa A., Tilli G., Piccirillo A. (2024). Development and Use of Biocheck.UGent^TM^ Scoring System to Quantify Biosecurity in Conventional Indoor (Turkey, Duck, Breeder) and Free-Range (Layer and Broiler) Poultry Farms. Prev. Vet. Med..

[97] Schreuder J., Simitopoulou M., Angastiniotis K., Ferrari P., Wolthuis-Fillerup M., Kefalas G., Papasolomontos S. (2023). Development and Implementation of a Risk Assessment Tool for Broiler Farm Biosecurity and a Health Intervention Plan in the Netherlands, Greece, and Cyprus. Poult. Sci..

